# Healing soles: a microbiology-driven electronic health record-algorithm and order set to decrease antipseudomonal use in diabetic foot infections, a retrospective, observational, quasi-experimental study

**DOI:** 10.1017/ash.2025.59

**Published:** 2025-03-27

**Authors:** Antoinette Marie Acbo, Naida Koura-Mola, Terrence McSweeney, Hongkai Bao, Mei Chang, Kelsie Cowman, Priya Nori, Yi Guo

**Affiliations:** 1 Department of Pharmacy, Montefiore Medical Center, Albert Einstein College of Medicine, Bronx, NY, USA; 2 Network Performance Group, Montefiore Medical Center, Albert Einstein College of Medicine, Bronx, NY, USA; 3 Division of Infectious Diseases, Montefiore Medical Center, Albert Einstein College of Medicine, Bronx, NY, USA

## Abstract

**Background:**

Antipseudomonal antibiotics are commonly prescribed for diabetic foot infections (DFI) at our institution despite a low local prevalence of *Pseudomonas aeruginosa*. A multidisciplinary team implemented a DFI electronic health record (EHR)-embedded treatment algorithm and order set.

**Methods:**

This multi-center, quasi-experimental study evaluated adults on antibiotics admitted for DFI to vascular surgery or medical units pre- and post-implementation of an EHR-embedded treatment algorithm and order set. Exclusion criteria: duplicate patients, concomitant infection, transfer from an outside hospital. Primary endpoint: antipseudomonal use among included patients (DOT/1000 DFI days present). Secondary outcomes: empiric antipseudomonal use, length of stay, 30-day readmission, mortality, amputation, and *Clostridioides difficile* infection. Patient demographics, diagnostics, treatments, and outcomes were evaluated.

**Results:**

Two hundred ten patients were included with 70 patients included in each group. The post-algorithm group had lower antipseudomonal DOT/1000 DFI days present compared to the pre-intervention group (360 vs 503, *P* < 0.001). The post-order set group had the lowest antipseudomonal use (347 vs 503, *P* < 0.001). Empiric antipseudomonal use decreased from 85.7% pre-intervention to 72% post-algorithm and 68.5% post-order set. Collectively, 30-day mortality was < 5%. Amputation during and within 30 days of hospitalization was similar in the pre-intervention (48.6%), post-algorithm (30%), and post-order set (41.4%) groups. Methicillin-susceptible *Staphylococcus aureus* and *Streptococcus* spp. were most frequently isolated. Wound cultures were not collected in 24.3%, 22.9%, and 40% of the pre-intervention, post-algorithm, and post-order set group.

**Conclusions:**

EHR-embedded clinical decision-making tools reduce antipseudomonal use for DFI treatment without increasing 30-day mortality or amputation.

## Introduction

Approximately 12% of the US population has diabetes and 13% of these patients suffer from diabetic foot ulceration. Diabetic foot infections (DFIs) contribute significantly to healthcare utilization.^1,2^ Between 2007 and 2013, approximately 4.2 million ambulatory care visits were for DFI.^3^ From 2006 to 2010, over 1 million cases of diabetic foot complications were presented to emergency departments in the United States.^4^ In all care settings, antimicrobial selection and optimization remains a pervasive challenge. The 2023 International Working Group on the Diabetic Foot/Infectious Diseases Society of America DFI treatment guidelines recommend against empiric pseudomonal coverage in patients without risk factors unless *Pseudomonas aeruginosa* was recently isolated from the site of infection.^5^ Previously identified risk factors include high local prevalence of *P. aeruginosa*, warm climate, and frequent water exposure.^6^ Prior studies found the incidence of pseudomonal DFI ranges from 4.5 to 9%.^7,8^ Despite low prevalence and DFI guideline recommendations, an estimated 88% of patients receive empiric antipseudomonal coverage.^7^

At Montefiore Medical Center, local prevalence of *P. aeruginosa* from DFI specimens was approximately 10% as determined by the microbiology department. Review of antimicrobial prescribing practices based on hospital unit and indication identified piperacillin/tazobactam as the most frequently prescribed agent for skin and soft tissue infections (SSTI) on several units treating DFI patients. Given the dissonance between high empiric antipseudomonal antibiotic use (AU) despite low local prevalence, two stewardship interventions were implemented to reduce antipseudomonal AU.

## Methods

### Study design

This retrospective, quasi-experimental study evaluated the efficacy of an EHR-embedded algorithm and order set. Data were collected during three periods: pre-implementation (12/1/2022-2/28/2023), post-algorithm implementation (12/1/2023-2/5/2024), and post-order set implementation (2/6/2024-5/1/2024). Participants and medication administrations were extracted using SAP BusinessObjects Business Intelligence Suite, a query system linked to the institution’s EHR. Patients identified via antibiotic administration, administration location, and medication indication data were screened for inclusion.

Patients were included if they were 18 years or older, admitted to a vascular surgery or medicine floor at Montefiore Medical Center (an academic medical center in Bronx, NY), and received antibiotic treatment for a DFI during their admission. Exclusion criteria included duplicate patients, concomitant infection unrelated to DFI, discharge against medical advice, or transfer from an outside hospital.

The study was reviewed and approved by Montefiore Medical Center’s institutional review board.

### Outcomes

The study’s primary outcome was antipseudomonal days of therapy (DOT) per 1000 days for included DFI patients. Antipseudomonal antibiotics included aztreonam, cefepime, ceftazidime/avibactam, ceftolozane/tazobactam, cefiderocol, ciprofloxacin, levofloxacin, meropenem, and piperacillin/tazobactam.

Secondary outcomes included empiric antipseudomonal AU, hospital length of stay (LOS), 30-day readmission, 30-day mortality, 30-day amputation, and 30-day *C. difficile* infection (CDI). Collected cultures were also evaluated.

### Statistical analysis

**Figure 1.**
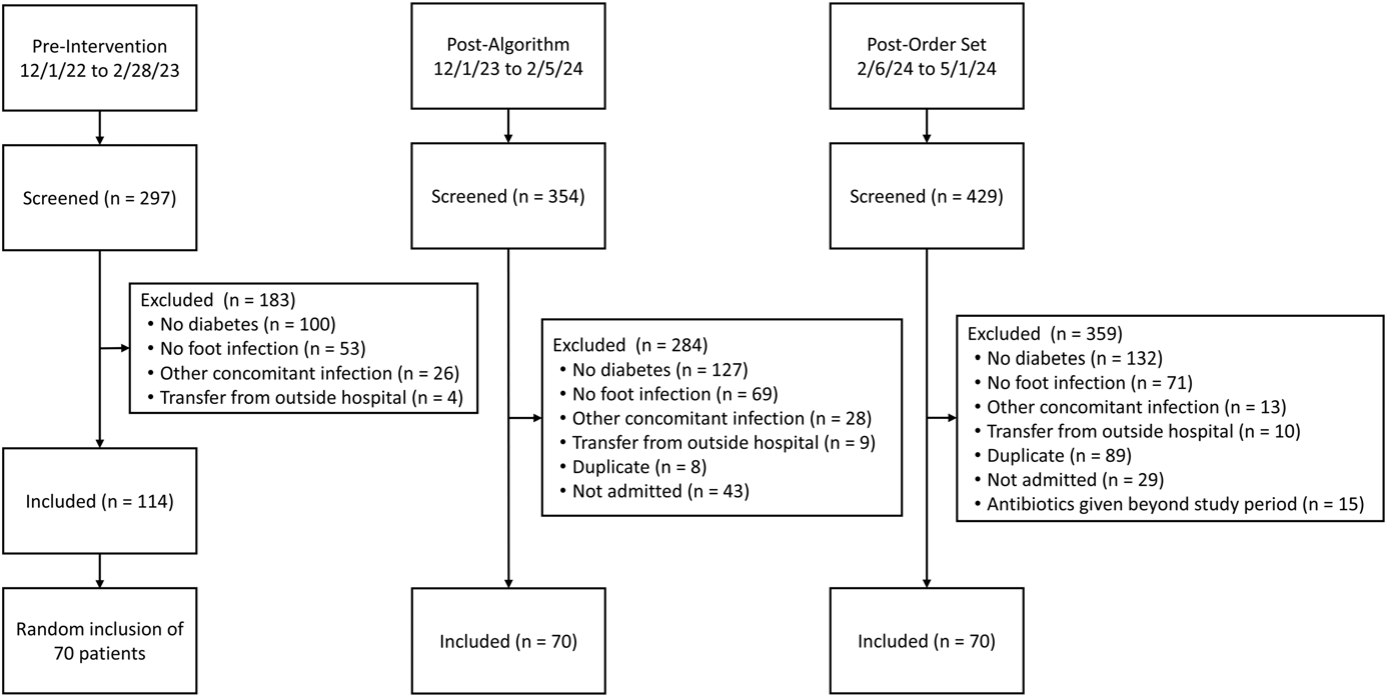
Inclusion and exclusion criteria.

Given prior literature findings of a decrease in empiric antipseudomonal antibiotic use from 64% to 40%,^9^ a sample size of 67 patients in each arm was calculated to detect a difference of this effect size with 80% power. Patients were identified and screened until 70 patients were included in each arm. The most common exclusion reason was no diabetes (Figure 1). Descriptive statistics were used to describe baseline demographics and secondary outcomes. Categorical data was analyzed using Chi-squared or Fisher’s exact test, as appropriate. Normally distributed continuous data was analyzed via student’s t-test and non-normally distributed continuous data was evaluated via Mann-Whitney-U test. Statistical significance was defined as p-value < 0.05.

### Interventions

Two clinical decision-making tools were implemented to optimize DFI treatment: an EHR-embedded algorithm (Supplementary Material 1) visible to all providers on the piperacillin/tazobactam ordering screen regardless of indication and a DFI order set (Supplementary Material 2). The order set included a group of bundled orders specific to the DFI syndrome such as recommended consults, labs, imaging, and antimicrobials. Development of both tools was led by infectious diseases (ID) pharmacists through the antimicrobial stewardship program (ASP) with input from ID, vascular surgery, emergency medicine (EM), and endocrinology providers. As part of a large health system, approval from affiliate institutions was required as EHR changes are implemented system-wide. The algorithm and order set were implemented on November 14, 2023 and February 6, 2024, respectively. After low order set uptake, the ASP provided additional education to EM and vascular surgery teams.

## Results

Two hundred ten patients were included. The post-algorithm group demonstrated a higher proportion of females compared to the pre-intervention group (Table 1). Notably, the pre-intervention group exhibited a higher proportion of Caucasian patients compared to post-intervention groups. Representing less than 15% of all groups, a small subset of immunocompromised patients was included. Pseudomonal risk factors were similar between the pre-intervention and post-intervention groups except for a higher rate of hospitalization 90 days prior to index visit in the post-algorithm group (10 (14.3%) vs 20 (28.6%), *P* = 0.04).

**Table 1.** Baseline characteristics

**Patient Characteristics**		**Pre-Intervention** **(n = 70)**	**Post-Algorithm** **(n = 70)**	**p-value**	**Post**- **Order Set** **(n = 70)**	**p-value**
Age, years, median (IQR)		65 (17)	59 (18)	0.04	67 (20)	0.98
Female sex, n (%)		21 (30)	22 (31.4)	0.85	24 (34.3)	0.59
BMI > 30 kg/m2, n (%)		18 (25.7)	25 (35.7)	0.2	26 (37.1)	0.15
Race, n (%)	Black	29 (41.4)	24 (34.3)	0.0003	27 (38.5)	0.0001
	White	25 (35.7)	9 (12.9)		5 (7.1)	
	Other/Declined	16 (22.9)	37 (52.8)		38 (54.2)	
Ethnicity, n (%)^a^	Non-Hispanic/Latino	36 (51.4)	32 (45.7)	0.30	35 (50)	0.60
	Hispanic/Latino	30 (42.9)	38 (54.2)		35 (50)	
Charlson Comorbidity Index, median (IQR)		5.5 (2.5)	4 (3)	0.33	5 (4)	0.44
Immunocompromised, n (%)		6 (8.6)	7 (10)	0.77	10 (14.2)	0.28
HgA1c, median % (IQR)		7.9 (2.28)	8.2 (3.0)	0.37	8.3 (2.8)	0.57
Hemoglobin at time of HgA1c, median (IQR)		11.1 (1.55)	11 (3.45)	0.53	10.8 (3.0)	0.62
Erythrocyte Sedimentation Rate, median (IQR)		73 (43)	71.5 (72.5)	0.45	75 (41)	0.90
C-Reactive Protein, median (IQR)		11.1 (1.8)	7.1 (10)	0.44	4.1 (10.7)	0.46
Pseudomonal Risk Factors, n (%)	IV Antibiotics, past 90 days	14 (20)	17 (24.3)	0.54	10 (14.3)	0.37
	Hospitalized, past 90 days	10 (14.3)	20 (28.6)	0.04	12 (17.1)	0.64
	*P. aeruginosa*, past 1 year	2 (2.9)	2 (2.8)	1	3 (4)	0.56
Severe DFI, n (%)		13 (18.6)	11 (15.7)	0.86	14 (20.0)	0.24
Antimicrobials held prior to surgical intervention or debridement, n (%)		6 (8.5)	2 (2.8)	0.15	5 (7.1)	0.75

BMI, body mass index; DFI, diabetic foot infection; HgA1c, hemoglobin A1c; IQR, interquartile range.

a Ethnicity was unknown in 4 patients pre-intervention, 0 post-algorithm, and 0 post-order set.

The most frequently isolated bacteria in the cultures collected were *Streptococcus* spp. (24.5% pre-intervention, 31.5% post-algorithm, and 4.8% post-order set) and methicillin-susceptible *S. aureus* (28.3% pre-intervention, 20.4% post-algorithm, and 33.3% post-order set). *P. aeruginosa* represented a small portion of collected isolates (9.4% pre-intervention, 11.1% post-algorithm, and 4.8% post-order set). Methicillin-resistant *S. aureus* occurred in 5.7%, 16.7%, and 7.1% of cultures collected pre-intervention, post-algorithm, and post-order set, respectively. Wound cultures were not collected in 24.3%, 22.9%, and 40% of the pre-intervention, post-algorithm, and post-order set group.

**Figure 2.**
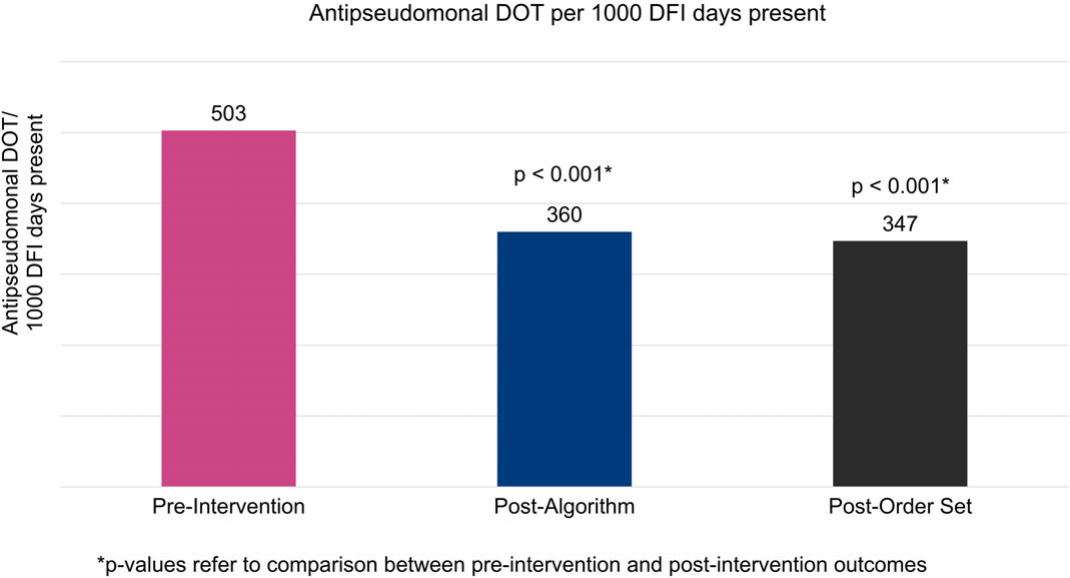
Antipseudomonal DOT (days of therapy)/1000 DFI (diabetic foot infection) days present during the pre-intervention (left), post-algorithm (center), and post-order set (right) periods.

Antipseudomonal use in included patients decreased significantly after implementation of the EHR-embedded algorithm (503 antipseudomonal DOT/1000 DFI days present vs. 360 antipseudomonal DOT/1000 DFI days present, *P* < 0.001). Post-order set, the difference remained significant (503 antipseudomonal DOT/1000 DFI days present vs. 347 antipseudomonal DOT/1000 DFI days present, *P* < 0.001) (Figure 2).

Empiric antipseudomonal use decreased post-algorithm (60 (85.7%) vs. 51 (72.8%), *P* = 0.06) and post-order set (60 (85.7%) vs 48 (68.5%), *P* = 0.01) (Table 2). Fewer amputations within 30 days occurred post-intervention compared to pre-intervention. The hospital LOS, 30-day readmission, and 30-day mortality rates were similar pre-intervention and post-implementation of the EHR-embedded algorithm and order set. There were no cases of CDI.

**Table 2.** Secondary outcomes

Outcomes	**Pre-Intervention** **(n = 70)**	**Post-Algorithm** **(n = 70)**	**p-value**	**Post-Order Set** **(n = 70)**	**p-value**
Empiric antipseudomonal antibiotic use, n (%)	60 (85.7)	51 (72.8)	0.06	48 (68.5)	0.01
Hospital length of stay, days, median (IQR)	7 (6)	7 (7)	0.31	7 (9)	0.74
30-day readmission, n (%)	15 (21.4)	15 (21.4)	1	16 (22.8)	0.84
30-day mortality, n (%)	3 (4.3)	2 (2.8)	0.65	1 (1.4)	0.31
Amputation within 30 d, n (%)	34 (48.6)	21 (30)	0.024	29 (41.4)	0.39
*C. difficile* infection within 30 d, n (%)	0 (0)	0 (0)	–	0 (0)	–


IQR, interquartile range.

## Discussion

Clinical decision support (CDS) tools are frequently utilized by ASPs. Active and passive interventions can improve patient outcomes, decrease antibiotic consumption, and narrow the spectrum of antibiotic usage.^10^ A prior study with a three-component stewardship intervention—DFI guidelines, an order set, and targeted education—demonstrated a significant decrease in antipseudomonal antibiotic use (538 DOT/1000 patient days to 272 DOT/1000 patient days).^9^

The implementation of the EHR-embedded algorithm to our institution’s piperacillin/tazobactam order screen was very effective. At our institution, EHR-embedded algorithm implementation was less laborious compared to order set implementation. Algorithms may be more accessible interventions to reduce antipseudomonal agent use compared to order sets. Both interventions require information technology support, but algorithms may require less development and implementation time. Order set use requires providers to be educated on its availability. The algorithm, however, was visible to all providers ordering piperacillin/tazobactam for any indication, creating more opportunities for use. Notably, addition of future algorithms to ordering screens may become cumbersome. Alternative CDS measures may be required pending EHR limitations.

Within our large health system, affiliate approval was required for both interventions. While health-system approval requires buy-in from various stakeholders, these interventions were widely accepted due to early interdisciplinary involvement with EM, endocrinology, vascular surgery, and ID providers and ubiquitous challenges with DFI treatment. Development of treatment recommendations based on local microbiology is an evidence-based approach to conducting syndrome-specific stewardship. Our ASP and microbiology colleagues conducted a five-year culture review of DFI ulcers collected from 2018 to 2022. Among 6,749 specimens from DFI-associated wound/abscess/tissue cultures, local prevalence of *P. aeruginosa* was approximately 10.4%. As acutely ill patients with severe SSTI are more likely to have cultures collected, 10.4% may represent an overestimate at our institution. Unit-specific AU data demonstrated high piperacillin/tazobactam use for SSTI. Providing this data to key stakeholders supported the interventions’ validity and helped obtain buy-in, a necessary step before information technology engagement.

This study has several limitations. The retrospective nature of chart review introduces potential bias and human error in data collection and interpretation. Reliance on existing medical records may include incomplete or inaccurate data entries. External data was not evaluated. Because the interventions were implemented with overlapping timelines, isolating intervention-specific impact is challenging. A lack of provider awareness resulted in low uptake of the DFI order set, and voluntary use limits its efficacy compared to a forced intervention. To increase order set use, targeted education with vascular surgery and EM providers was provided periodically, and CDS was encouraged among ID and stewardship teams. Ongoing evaluation of the algorithm and order set is necessary to ensure sustainability and optimize use for providers. Exclusion of patients with concomitant infections, like COVID-19 or influenza, may have influenced clinical presentation but were not expected to affect antimicrobial agent selection. Lastly, while DFIs are frequently treated outpatient,^3^ this study evaluated implementation and outcomes within the inpatient setting. Pseudomonal risk factors and general principles of antibiotic selection are not expected to vary significantly between settings.

This study supports utilization of an EHR-embedded algorithm and order set, as supported by local microbiology data, to decrease inpatient antipseudomonal antimicrobial use without increasing mortality or amputation risk. Despite recommendations in the algorithm and order set to hold antimicrobials before debridement or surgical intervention, most patients received empiric antimicrobial therapy. Data suggests empiric antimicrobial use is associated with increased risk of hospitalization compared to culture-directed antibiotic use in outpatient settings.^11^ Further interventions may reinforce the benefits of culture collection prior to antimicrobial use to medical and surgical teams.

Based on prior and current findings, reduced antibiotic consumption is a positive result of CDS (treatment protocols, algorithms, order sets, and team education on CDS tools) as encouraged and supported by the ASP. These interventions’ success enabled our ASP to proceed with additional system-wide, local microbiology-based CDS for other common infections, such as intra-abdominal infections and ventilator-associated pneumonia.

In conclusion, an EHR-embedded treatment algorithm and order set significantly reduced antipseudomonal antibiotic use at a large, urban, academic medical center without impact on 30-day mortality or 30-day amputation rates.

## Supporting information

Acbo et al. supplementary material 1 Acbo et al. supplementary material

Acbo et al. supplementary material 2 Acbo et al. supplementary material

## Supplementary material

The supplementary material for this article can be found at https://doi.org/10.1017/ash.2025.59
