# Supplementary figures and images for: Healing soles: a microbiology-driven electronic health record-algorithm and order set to decrease antipseudomonal use in diabetic foot infections, a retrospective, observational, quasi-experimental study
Source: Antimicrob Steward Healthc Epidemiol. 2025 Mar 27;5(1):e89. doi: 10.1017/ash.2025.59 (PMC11951233; doi:10.1017/ash.2025.59)

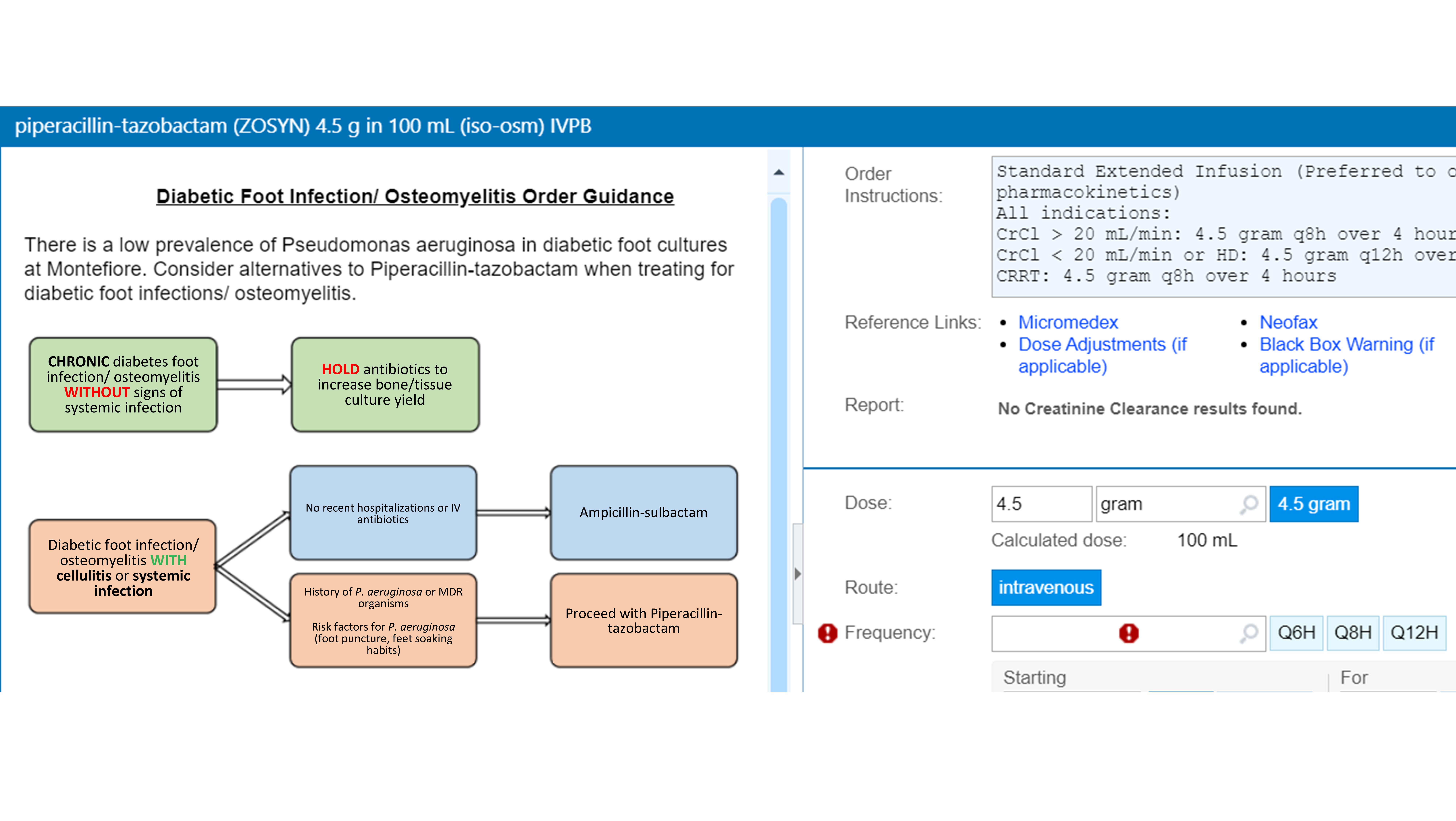

Supplement: Acbo et al. supplementary material 1 — Acbo et al. supplementary material [file S2732494X25000592sup001.tif]
