# Supplementary material for: Healing soles: a microbiology-driven electronic health record-algorithm and order set to decrease antipseudomonal use in diabetic foot infections, a retrospective, observational, quasi-experimental study
Source: Antimicrob Steward Healthc Epidemiol. 2025 Mar 27;5(1):e89. doi: 10.1017/ash.2025.59 (PMC11951233; doi:10.1017/ash.2025.59)
Supplement: Acbo et al. supplementary material 2 — Acbo et al. supplementary material [file S2732494X25000592sup002.pdf]

Diabetic Foot Infection Including Osteomyelitis

Manage User Versions | Remove Order Sets

Disclaimer: If patient meets criteria for sepsis, please order through "Sepsis/ Septic Shock pathway"

General

Activity

- [ ] Weight bearing as tolerated
- [ ] Non weight bearing

Consults

- [ ] Inpatient consult to vascular surgery
- [ ] Inpatient consult to Endocrinology
- [ ] Inpatient consult to Infectious Diseases
- [ ] Inpatient consult to Case Management
- [ ] Inpatient consult to social work
- [ ] Inpatient consult to wound care

Labs

Labs and Diagnostics

- [ ] Basic Metabolic Panel
- [ ] CBC WITH DIFFERENTIAL
- [ ] Nasal, MRSA Screen by PCR
- [ ] Erythrocyte Sedimentation Rate
- [ ] C-Reactive Protein (CRP)
- [ ] Hemoglobin A1c
- [ ] Point of care Glucose

Imaging

Imaging

- [x] X-ray Orders
  - [ ] XR FOOT - ROUTINE - 3 VIEWS - PA LATERAL AND OBLIQUE - LEFT
  - [ ] XR FOOT - ROUTINE - 3 VIEWS - PA LATERAL AND OBLIQUE - RIGHT
- [ ] MRI Orders
- [ ] Abi & Pvr Lower Extremity Complete Bilat

Medications

Antibiotics

(●) Chronic diabetic foot infection/ osteomyelitis WITHOUT evidence of cellulitis or systemic signs of infection

IF patient is planned for surgical intervention in the next 48-72h:

Hold antibiotics to increase bone/tissue culture yield and help guide therapy. Start empiric antibiotics if patient develops worsening infection (i.e. increased erythema, purulent discharge, fever, leukocytosis), or if significant delay in surgical intervention is expected.

(●) No plans for surgical intervention/conservative management

Consider oral therapy and outpatient ID follow up

No recent antibiotic exposure:

- Amoxicillin/clavulanate 875 mg PO q12h (adjust dose and frequency based on renal function) OR
- Cephalexin 500 mg PO q6h (adjust frequency based on renal function) OR
- Cefuroxime 500 mg PO q12h (adjust frequency based on renal function)

Recent antibiotic exposure:

- Levofloxacin 750 mg PO q24h (adjust frequency based on renal function) OR
- Cefdinir 300 mg PO q12h (adjust frequency based on renal function)

High risk for MRSA, add:

- Doxycycline 100 mg PO BID OR
- Trimethoprim/sulfamethoxazole 1-2 DS tabs PO BID (adjust based on weight and renal function)

- [ ] amoxicillin-clavulanate (AUGMENTIN) 875-125 mg per tablet
  1 tablet, 2 (two) times a day
- [ ] amoxicillin-clavulanate (AUGMENTIN) 500-125 mg per tablet
  1 tablet, 2 (two) times a day
- [ ] cephalexin capsule
  500 mg
- [ ] cefUROXime tablet
  500 mg, 2 (two) times a day
- [ ] cefdinir (OMNICEF) capsule
  300 mg, 2 (two) times a day
- [ ] levoFLOXacin tablet
  750 mg
- [ ] doxycycline tablet
  100 mg, 2 (two) times a day
- [ ] sulfamethoxazole-trimethoprim (BACTRIM DS) 800-160 mg per tablet

( ) Diabetic foot infection/ osteomyelitis WITH evidence of cellulitis or systemic signs of infection

Medications

Antibiotics

- ( ) Chronic diabetic foot infection/ osteomyelitis WITHOUT evidence of cellulitis or systemic signs of infection
- (•) Diabetic foot infection/ osteomyelitis WITH evidence of cellulitis or systemic signs of infection
  - (•) ! No ABX exposure within the past 90 days, no history of pseudomonas or MDR organisms

    Recommended regimens:

    - Ampicillin-sulbactam 3 g IV q6h (adjust frequency based on renal function) OR
    - Cefazolin 2 g IV q8h* (adjust dose and frequency based on renal function) OR
    - Ceftriaxone 1 – 2 g IV q24h* (2 g dose recommended for OM and when BMI > 30)
    - ADD vancomycin for patients with MRSA risk factors
    - *ADD metronidazole 500 mg PO q12h (IV if patient NPO) ONLY to cephalosporin regimens for patients with ischemic limb/ necrosis/ gas-forming infections

    - [ ] ampicillin-sulbactam (UNASYN) IV
      3 gram
    - [ ] ceFAZolin IV
    - [ ] cefTRIAXone IV
      every 24 hours as directed
    - [ ] metroNIDAZOLE tablet
      500 mg, every 12 hours
    - [ ] metroNIDAZOLE IV
      500 mg, every 12 hours
    - [ ] vancomycin IV
  - ( ) History of Pseudomonas aeruginosa or MDR organisms, risk factors for Pseudomonas aeruginosa (foot puncture, water exposure, or recent IV antibiotics)
  - ( ) Anaphylaxis to penicillin or cephalosporin

Medications

Antibiotics

- ( ) Chronic diabetic foot infection/ osteomyelitis WITHOUT evidence of cellulitis or systemic signs of infection
- (•) Diabetic foot infection/ osteomyelitis WITH evidence of cellulitis or systemic signs of infection
  - ( ) No ABX exposure within the past 90 days, no history of pseudomonas or MDR organisms
  - (•) ! History of Pseudomonas aeruginosa or MDR organisms, risk factors for Pseudomonas aeruginosa (foot puncture, water exposure, or recent IV antibiotics)

    Recommended regimens:

    - Piperacillin tazobactam 4.5 g IV q8h run over 4 hours (adjust frequency for 30-minute infusions and renal function) OR
    - Cefepime 2 g IV q8h* (adjust dose and frequency based on renal function)
    - ADD vancomycin for patients with MRSA risk factors
    - *ADD metronidazole 500 mg PO q12h (IV if patient NPO) ONLY to cephalosporin regimens for patients with ischemic limb/ necrosis/ gas-forming infections

    - [ ] piperacillin-tazobactam (ZOSYN) IV
      4.5 gram
    - [ ] cefepime IV
      2 gram
    - [ ] metroNIDAZOLE tablet
      500 mg, every 12 hours
    - [ ] metroNIDAZOLE IV
      500 mg, every 12 hours
    - [ ] vancomycin IV
  - ( ) Anaphylaxis to penicillin or cephalosporin

Medications

Antibiotics

- ( ) Chronic diabetic foot infection/ osteomyelitis WITHOUT evidence of cellulitis or systemic signs of infection
- (•) Diabetic foot infection/ osteomyelitis WITH evidence of cellulitis or systemic signs of infection
  - ( ) No ABX exposure within the past 90 days, no history of pseudomonas or MDR organisms
  - ( ) History of Pseudomonas aeruginosa or MDR organisms, risk factors for Pseudomonas aeruginosa (foot puncture, water exposure, or recent IV antibiotics)
  - (•) ! Anaphylaxis to penicillin or cephalosporin

    Recommended regimens:

    - Aztreonam 2 g IV q8h (adjust based on renal function) + vancomycin OR
    - Levofloxacin 750 mg PO/IV q24h (adjust dose and frequency based on renal function) + vancomycin (for patients with MRSA risk factors)
    - ADD metronidazole 500 mg PO q12h (IV if patient NPO) for patients with ischemic limb/ necrosis/ gas-forming infections

    - [ ] aztreonam IV
      2 gram
    - [ ] vancomycin IV
    - [ ] levoFLOXacin tablet
      750 mg
    - [ ] levoFLOXacin IV
      750 mg
    - [ ] metroNIDAZOLE tablet
      500 mg, every 12 hours
    - [ ] metroNIDAZOLE IV
      500 mg, every 12 hours
